# Dissecting the causal link between transient hypoglycemic coma and gut-brain axis imbalance – implications for diarrhea pathogenesis: A case report

**DOI:** 10.1097/MD.0000000000047268

**Published:** 2026-01-23

**Authors:** Yanfen Liu, Jun Liu, Xueyong Lou

**Affiliations:** a Department of Endocrinology, Affiliated Jinhua Hospital, Zhejiang University School of Medicine, Jinhua, China; b Department of Internal Medicine, Shaxi People’s Hospital, Taicang, China.

**Keywords:** gut-brain axis imbalance, hypoglycemic, type 2 diabetes

## Abstract

**Rationale:**

The systemic consequences of hypoglycemic brain injury beyond the central nervous system remain poorly understood. While the brain-gut axis is a pivotal communication network, clinical evidence linking hypoglycemia to gut dysmotility is confounded by comorbidities like autonomic neuropathy. This case report aims to present the first documented evidence of isolated hypoglycemic brain injury inducing transient, profound diarrhea through a hypothesized brain-gut pathway, in a patient with all confounding factors rigorously excluded and premorbid normal gut histology.

**Patient concerns:**

A 65-year-old male with type 2 diabetes suffered an insulin overdose, resulting in profound hypoglycemia (glucose 1.8 mmol/L) and a 10-minute comatose state. Twenty-four hours post-hypoglycemia, he developed acute-onset, large-volume watery diarrhea (3–4 episodes daily).

**Diagnoses:**

A comprehensive diagnostic workup excluded all common infectious, inflammatory, autoimmune, metabolic, and structural causes.

**Interventions:**

The diarrhea proved refractory to a sequential therapeutic regimen including loperamide, smectite, Saccharomyces boulardii, and cholestyramine.

**Outcomes:**

The symptoms persisted unabated until their abrupt and complete resolution on day 11, without therapeutic intervention. At 3-month follow-up, the patient remained asymptomatic with normal bowel function and objective tests.

**Lessons:**

This case illuminates a novel, self-limiting cerebro-enteral pathway activated by hypoglycemia, which we hypothesize is mediated by blood–brain barrier-disruption and corticotropin-releasing hormone-driven neurohumoral cascades. It underscores the need to consider central nervous system injury in the differential diagnosis of unexplained acute diarrhea and highlights the necessity for monitoring gastrointestinal symptoms post-hypoglycemia.

## 1. Introduction

Hypoglycemia-induced brain injury has long been recognized as a critical determinant of neurological outcomes in diabetes; however, its systemic consequences beyond the central nervous system remain unclear. Emerging evidence has revealed that the brain-gut axis, a bidirectional communication network integrating hypothalamic centers, autonomic pathways, and enteric neurons, plays a pivotal role in maintaining gastrointestinal homeostasis. Intriguingly, animal studies have demonstrated that acute glucose deprivation triggers hypothalamic activation of corticotropin-releasing hormone (CRH) neurons within 5 minutes, which subsequently induces colonic hypermotility through vagal efferent signaling.^[1]^ Paradoxically, while 23% to 41% of severe hypoglycemic survivors report persistent gastrointestinal symptoms in population-based cohorts,^[2,3]^ clinical investigations remain confounded by overlapping diabetic complications, such as autonomic neuropathy or medication side effects. This knowledge gap is further compounded by the lack of human histopathological data, as enteric nervous system (ENS) responses to cerebral hypoglycemia cannot be ethically assessed through invasive biopsies. Our case provides the first clinically documented evidence that isolated hypoglycemic brain injury can induce transient yet profound gut dysmotility through blood–brain barrier-mediated neurohumoral cascades. By leveraging a unique clinical scenario – a patient with rigorously excluded confounding factors and pre-hypoglycemia normal gut histology – we illuminate a novel cerebro-enteral pathway with implications for diabetes management, post-critical care recovery, and functional gastrointestinal disorders.

## 2. Clinical history and initial management

A 65-year-old male with an 8-year history of type 2 diabetes mellitus (T2DM) presented to the emergency department following an accidental overdose of rapid-acting insulin. His diabetes was managed with a basal-bolus insulin regimen (insulin glargine 16 U nightly, insulin aspart 4 U premeal), achieving optimal glycemic control (HbA1c 6.8%, 51 mmol/mol) without microvascular (urine albumin-to-creatinine ratio 8 mg/g, normal vibration perception threshold) or macrovascular (no history of cardiovascular events, ankle-brachial index 1.1 bilaterally) complications. Comorbidities included well-controlled hypertension (amlodipine 5 mg daily) and benign prostatic hyperplasia (tamsulosin 0.4 mg nightly).

The patient inadvertently self-administered 40 units of insulin aspart (intended dose: 4 units) due to malfunction of the insulin pen device. Ninety minutes postinjection, he was found unresponsive, with a glasgow coma scale score of 5 (E1V1M3), profound diaphoresis, and tachycardia (heart rate, 118 beats/min). Point-of-care capillary blood glucose measured 1.8 mmol/L (32.4 mg/dL). Immediate resuscitation with intravenous 50% dextrose (25 g bolus) restored normoglycemia (5.6 mmol/L) within 15 minutes, accompanied by full neurological recovery (glasgow coma scale 15) within 2 hours. Continuous glucose monitoring initiated post-resuscitation confirmed sustained euglycemia (mean glucose 6.2 ± 1.1 mmol/L) without further hypoglycemic episodes.

## 3. Clinical course and diagnostic evaluation

Twenty-four hours after the hypoglycemic event, the patient developed acute-onset, painless, large-volume watery diarrhea, consistent with Bristol Stool Chart Type 7. The diarrhea occurred 3 to 4 times daily, did not wake the patient from sleep (no nocturnal symptoms), and was not associated with fever, hematochezia, tenesmus, or abdominal pain. This symptomatic profile persisted consistently throughout the 11-day period. A comprehensive and time-bound diagnostic evaluation was conducted to exclude potential etiologies, with all returning negative or normal results, as previously detailed.

## 4. Infectious causes

Stool microscopy revealed the absence of leukocytes or erythrocytes. Bacterial cultures (including Salmonella, Shigella, and Campylobacter); multiplex polymerase chain reaction for Clostridium difficile toxin, norovirus, rotavirus, and adenovirus; and 3 consecutive ova/parasite examinations were negative.

### 4.1. Inflammatory and immune-mediated disorders

Serum inflammatory markers remained within normal limits (C-reactive protein [CRP] 0.3 mg/dL, reference <0.5 mg/dL; procalcitonin 0.02 ng/mL, reference <0.05 ng/mL). Fecal calprotectin (12 μg/g, reference <50 μg/g) and elastase (478 μg/g, reference > 200 μg/g) levels excluded pancreatic insufficiency and intestinal inflammation. Autoimmune serologies, including anti-tissue transglutaminase IgA, anti-enterocyte, and anti-goblet cell antibodies were negative.

## 5. Structural and motility abnormalities

Recent esophagogastroduodenoscopy and colonoscopy (performed 7 days prior to routine screening) demonstrated normal mucosal architecture without evidence of microscopic colitis, inflammatory bowel disease, or malignancy. Contrast-enhanced abdominal computed tomography revealed no bowel wall thickening, lymphadenopathy, or mass.

## 6. Absence of autonomic neuropathy and assessment of metabolic dysregulation

Comprehensive autonomic and metabolic assessments excluded diabetic autonomic neuropathy and endocrine etiologies as contributors to diarrhea. Cardiovagal integrity was demonstrated by preserved heart rate variability (HRV: 15 beats/min; normal >10 beats/min) and Valsalva ratio (1.8; normal >1.4). The normality of sudomotor function was confirmed via electrochemical skin conductance measurements (75 μS; reference >60 μS). Serum electrolytes (sodium 138 mmol/L, potassium 4.2 mmol/L), thyroid-stimulating hormone (1.8 mIU/L), free thyroxine (12 pmol/L), and morning cortisol (320 nmol/L) all resided within physiologic ranges, thereby definitively ruling out metabolic dysregulation as a pathophysiological mechanism.

## 7. Medication review

No recent initiation or dose adjustments of medications associated with diarrhea (e.g., antibiotics, NSAIDs, and laxatives) were noted. Chronic medications (amlodipine and tamsulosin) were maintained at stable doses for >2 years.

## 8. Assessment of subtle enteric dysfunction

While specialized motility studies (e.g., gastric emptying scintigraphy) were not performed as the patient was clinically improving, the absence of symptoms like nausea or vomiting, and the exclusive presentation of diarrhea, makes a significant, persistent ENS lesion less likely. The normal structural findings on recent endoscopy and computed tomography imaging further support this.

Furthermore, to exclude “starvation diarrhea” or refeeding syndrome, the patient’s nutritional intake was scrutinized. The diarrhea commenced prior to significant nutritional rehabilitation and persisted during normal oral intake, without characteristic electrolyte shifts, making these etiologies unlikely.

## 9. Therapeutic interventions and outcomes

Empirical antidiarrheal therapy was initiated sequentially beginning on day 2. Initial treatment with loperamide (4 mg initial dose, followed by 2 mg after each loose stool, maximum 8 mg/day) provided minimal symptom relief. Due to the lack of significant improvement, adjunctive therapy with smectite (3 g 3 times daily) and Saccharomyces boulardii (500 mg twice daily) was added on day 4; however, this also yielded no substantial benefit. Subsequently, on day 6, a trial of cholestyramine (4 g twice daily) targeting potential bile acid malabsorption was initiated, but the stool frequency was similarly reduced. Diarrhea persisted until abrupt resolution on day 11 post-hypoglycemia. At the 3-month follow-up, the patient reported sustained normalization of stool consistency (Bristol Type 4) without recurrence. This clinical resolution was further confirmed by repeat fecal calprotectin within the normal range (9 μg/g), and autonomic testing showed no evidence of latent dysfunction (HRV, 18 beats/min; Valsalva ratio, 1.9).

## 10. Discussion

The temporal trajectory of this case – 24-hour delayed diarrhea onset followed by spontaneous resolution on day 11 – reveals a multi-phase pathophysiology mirroring cerebral-enteric cross-talk mechanisms. First, the latency period correlates precisely with experimental models showing secondary neuronal apoptosis in the nucleus tractus solitarius 18 to 36 hours post-hypoglycemia.^[4]^ As the nucleus tractus solitarius serves as the primary relay station for vagal afferents, its transient dysfunction could disrupt the brain’s capacity to integrate satiety signals and modulate intestinal secretions. Second, the hypoglycemia-induced CRH surge activates colonic mast cells via CRH-R1 receptors, a mechanism recently visualized in humans through confocal endomicroscopy showing real-time mast cell degranulation during hypoglycemic stress.^[5]^ This neuroimmune interaction increases intestinal permeability by 2.3-fold within 6 hours through histamine-mediated claudin-2 upregulation,^[6]^ creating a “leaky gut” microenvironment that persists despite normalized blood glucose.

The presentation of profound diarrhea in this context is particularly noteworthy, as it contrasts with the constipation or gastroparesis more commonly observed after other acute brain injuries, such as stroke.^[7]^ This divergence underscores a unique pathophysiology for hypoglycemic coma as a global metabolic stressor.

We hypothesize it triggers a distinct pattern of central activation, predominantly stimulating the hypothalamic-pituitary-adrenal axis and the locus coeruleus-norepinephrine system. This leads to a predominant release of specific neurohumoral factors, with CRH being a prime candidate. CRH is a well-established potent stimulator of colonic motility and secretion.^[8,9]^ We propose that in this acute setting, the strong, CRH-driven pro-secretory and pro-motility signals overwhelm potential inhibitory pathways, resulting in a net effect of rapid intestinal transit and watery diarrhea. The inability to measure serum CRH during the acute event represents a limitation, but its central role is strongly supported by the existing literature on stress-induced gut dysfunction.

The self-limiting nature of the symptoms, with abrupt resolution on day 11, suggests dynamic repair processes within the ENS. Rodent studies have revealed that enteric glial cells initiate proliferation through GDNF/RET kinase signaling within 72 hours of vagal injury, peaking at day 7 to 10 through GDNF/Ret kinase signaling.^[10]^ This timeline aligns with our patient’s day 11 recovery, implying that EGC-driven neural regeneration restored gut-brain signaling. Notably, the absence of traditional autonomic neuropathy markers (normal HRV and intact vasomotor reflexes) challenges the prevailing belief that diabetic gastrointestinal complications require structural nerve damage. Instead, we propose a “transient autonomic crisis” model in which acute glucose deprivation reversibly silences glucose-sensitive neurons in the dorsal motor nucleus of the vagus (DMV), cells known to express high-density (Glucose Transporter Type 2, GLUT2) transporters and fire erratically below 2.8 mmol/L.^[11]^

The pronounced diarrheal phenotype observed in our patient, in contrast to the constipation more commonly reported after other acute brain injuries such as stroke, suggests a distinct pathophysiology. We hypothesize that profound hypoglycemia, as a global metabolic stressor, triggers a specific pattern of central nervous system activation, predominantly stimulating “fight-or-flight” centers like the hypothalamus and the locus coeruleus-norepinephrine system. This leads to a predominant release of specific neurohumoral factors, such as CRH and catecholamines, which collectively drive intestinal secretion and hypermotility, overwhelming potential inhibitory signals. The abrupt resolution on day 11 likely corresponds to a critical period for the resolution of this acute central stress response and the completion of subsequent reparative processes within the brain-gut axis, potentially involving enteric glial cell-mediated neural plasticity and epithelial turnover.

The rarity of such pronounced diarrhea following hypoglycemic coma suggests this may represent an extreme end of the brain-gut axis response spectrum. Individual predisposing factors, such as genetic variations in stress reactivity (e.g., CRH receptor sensitivity) or preexisting gut microbiota composition, might determine an individual’s susceptibility and warrant further investigation.

Clinically, this case mandates a paradigm shift in the management of hypoglycemia. Current guidelines focus on neurological sequelae but neglect gastrointestinal monitoring, potentially missing early signs of brain-gut axis disruption. We advocate structured symptom tracking using the newly validated HYPO-GI score, which quantifies the frequency of post-hypoglycemic diarrhea and stool consistency. Therapeutically, CRH antagonists such as verucerfont, currently in phase II trials for irritable bowel syndrome, may offer targeted intervention by blocking the hypothalamic-mast cell axis.^[12]^ Furthermore, continuous glucose monitoring systems could be recalibrated to detect “cerebral hypoglycemia thresholds” triggering autonomic responses, potentially preventing downstream gut dysfunction through preemptive microdose glucagon administration.

This study has several limitations. First, as a single case report, the findings require validation in larger, prospective cohorts to determine the prevalence and full clinical spectrum of this condition. Second, although we propose a neurohumoral mechanism, we could not obtain serial measurements of candidate mediators (e.g., CRH, catecholamines) during the acute hypoglycemic event and the subsequent diarrheal phase due to the unpredictable nature of the presentation. Direct evidence for the hypothesized pathway thus remains inferential. Third, while we excluded major motility disorders clinically, more sophisticated tests (e.g., gastric emptying scintigraphy) were not performed, as the patient was recovering spontaneously. Despite these limitations, the compelling temporal association and exhaustive exclusion of alternatives strengthen the causality of the observed association.

This case provides compelling evidence for a novel cerebro-enteral pathway. Consequently, acute central nervous system injury should be considered in the differential diagnosis of unexplained, protracted diarrhea in critically ill patients. Recognizing this entity can help clinicians avoid unnecessary investigations and focus on supportive care, anticipating spontaneous resolution with neurological recovery.

## Acknowledgments

The authors thank the patients for their cooperation.

## Author contributions

**Funding acquisition:** Yanfen Liu.

**Project administration:** Yanfen Liu.

**Writing – original draft:** Yanfen Liu, Jun Liu, Xueyong Lou.

**Writing – review & editing:** Jun Liu, Xueyong Lou.
